# Enhancing rapid Protein A performance in monoclonal antibody processing: Anion exchange chromatographic clarification

**DOI:** 10.1002/btpr.70061

**Published:** 2025-08-07

**Authors:** Andrew Vail, David Chau, Jennifer Heitkamp, Alexei Voloshin

**Affiliations:** ^1^ Purification and Filtration Business Solventum St. Paul Minnesota USA

**Keywords:** anion exchange, bioprocessing, chromatographic clarification, clarification, impurity reduction, Protein A chromatography

## Abstract

Clarification fidelity, including reduction of insoluble and soluble contaminants, has been demonstrated to significantly affect the performance and robustness of the Protein A capture chromatography step during the purification of monoclonal antibodies (mAb) and their derivatives expressed in CHO cell cultures. While the vast majority of previous studies have focused on the evaluation of these effects on conventional Protein A resins, in this study, we evaluated such effects on the new membrane‐ and fiber‐based Protein A technologies. Both depth filtration and chromatographic clarification using charged functional fiber approaches have been studied, and we evaluated the effects of these methods on convective Protein A technology cycling robustness, as well as the purity of the product in the elution pool with respect to process‐related contaminants. We found that clarification of CHO cell culture using anion exchange (AEX) fiber significantly increases the purity of the mAb in the elution pool with respect to host cell protein (at least 50% less) and DNA (>2 log less) as well as enables a higher number of Protein A cycles (at least 2X increase in fiber‐based Protein A cycling lifetime) compared to CHO cell culture fluid clarified with conventional depth filtration. It is likely that this is due to superior DNA and sub‐500 nm particle reduction during the chromatographic fiber clarification. This work elucidates the importance of a holistic process strategy when designing a biopharmaceutical purification process.

## 1 INTRODUCTION

Process simplification and intensification are the two defining megatrends in the bioprocessing space today. As biotherapeutic candidate pipelines continue to grow, the need for productive and scalable manufacturing platforms has never been more urgent. Monoclonal antibodies (mAbs) and their derivatives continue to drive the recombinant protein therapeutic space and are projected to do so for the foreseeable future. As such, the mAb manufacturing platform continues to be improved at all stages and operations. More and more holistic approaches are being deployed to create advanced processing strategies to increase productivity and to reduce the cost of mAb manufacturing. One of the more significant examples has been the move of anion exchange (AEX) chromatographic separations, further upstream, into product harvest and clarification unit operation using AEX functional fiber technologies. Performing AEX separation during clarification simplifies and streamlines the mAb bioprocess by enabling higher reduction of soluble and insoluble contaminants very early in the process.^1^, ^2^, ^3^, ^4^, ^5^, ^6^, ^7^

Protein A resin utilization has dominated the recently published literature since convective Protein A technologies based on fiber or membrane supports are in their infancy, both in the academic and industrial space. Several non‐resin Protein A stationary phases have been reported in the literature, several commercially available, and consisting of membranes, monoliths, and fibers.^8^, ^9^, ^10^, ^11^, ^12^, ^13^, ^14^ Besides distinct structural differences with the stationary phase, these technologies are fundamentally different from resin as the Protein A ligand may be located on the surface of a fiber or a relatively large pore. The location of the ligand has more exposure to components of a mAb containing clarified cell culture fluid (CCCF) compared to a resin, where the ligand is better protected from larger particles (e.g., host cell DNA) by a pore diffusional barrier. Higher exposure to CCCF components can lead to higher impurity carryover and lower lifetime use. As the industry moves to examine and implement such technologies, it is critical to understand if process strategies to extend lifetime use and performance observed using Protein A resins are applicable to these new convective capture technologies.

The aim of this study was to identify the benefits of a fiber‐based chromatographic clarification strategy on the performance of convective Protein A solutions. Over the last several years, reported literature indicated that the use of fiber AEX chromatography during clarification unit operations significantly improved Protein A resin performance in terms of host cell proteins (HCP) and host cell DNA (HC‐DNA) clearance and fouling over the course of cycling. Clarification of cell culture using an AEX functionalized nonwoven media, 3M™ Emphaze™ AEX Hybrid Purifier, showed Protein A chromatography was 20 times more effective at HCP reduction.^2^ In another study, AEX chromatographic clarification demonstrated reduction of the HC‐DNA, rather than HCP, enabled higher performance of the Protein A capture step.^3^ Additionally, AEX functional fiber clarified feed achieved less overall fouling of a Protein A resin column cycled 100 times compared to depth filter clarified feed.^15^

In the past 20 years, convective chromatography technologies have been developed and commercialized because of the need for faster and more flexible processing. Here we examine the feed quality generated by AEX fiber chromatographic clarification and its impact on new non‐resin Protein A formats. This study further elaborates on how the newer convective‐based Protein A formats can be utilized with the benefits provided from AEX chromatographic clarification. Performing a basic Protein A cycling protocol with different CCCF examined feed quality on lifetime and purity of recovered product during capture cycling of two types of rapid Protein A laboratory devices.

## 2 MATERIALS AND METHODS

### 2.1 Materials

0.1 and 1 M sodium hydroxide were sourced from J.T. Baker. Tris base was sourced from Fisher Bioreagents. Sodium chloride was sourced from Fisher Chemicals. Gibco 1X PBS was sourced from Life Technologies. Ultrapure 1 M Tris–HCl, pH 7.5 was sourced from Invitrogen. Acetic acid was purchased from Supelco (Millipore). Guanidine hydrochloride was purchased from Sigma Aldrich. Nalgene Rapid‐Flow bottle top filters (0.2 μm, aPES) were purchased from Thermo Scientific. Sartopore® 2 filters were purchased from Sartorius Stedim. 3M™ Zeta Plus™ Depth Filter Capsules (E0340FSA90SP08A, E0340FSA90ZB08A) and 3M™ Emphaze™ AEX Hybrid Purifier (EMP513AEX020R) were from 3M Corporation. HiTrap Fibro™ PrismA was obtained from Cytiva. GORE® Protein Capture Device with Protein A columns was purchased from W. L. Gore & Associates. Chromatography experiments were conducted on an AKTA™ Pure 150 (Cytiva).

### 2.2 Cell culture production and centrifugation

Chinese hamster ovary (CHO) cell cultures expressing a biosimilar therapeutic monoclonal antibody (isoelectric point 8.6) were used for this study. Two separate cell cultures were produced in a Wave bioreactor (Cytiva) in fed‐batch mode producing an IgG1 to titers of 3.6 g/L and 4 g/L. At the time of harvest, cell culture fluid was processed through a disk‐stack centrifuge (Whisperfuge® by GEA). The centrate produced for each culture was aliquoted and stored at −30°C until further processing.

### 2.3 Clarification

**FIGURE 1:**
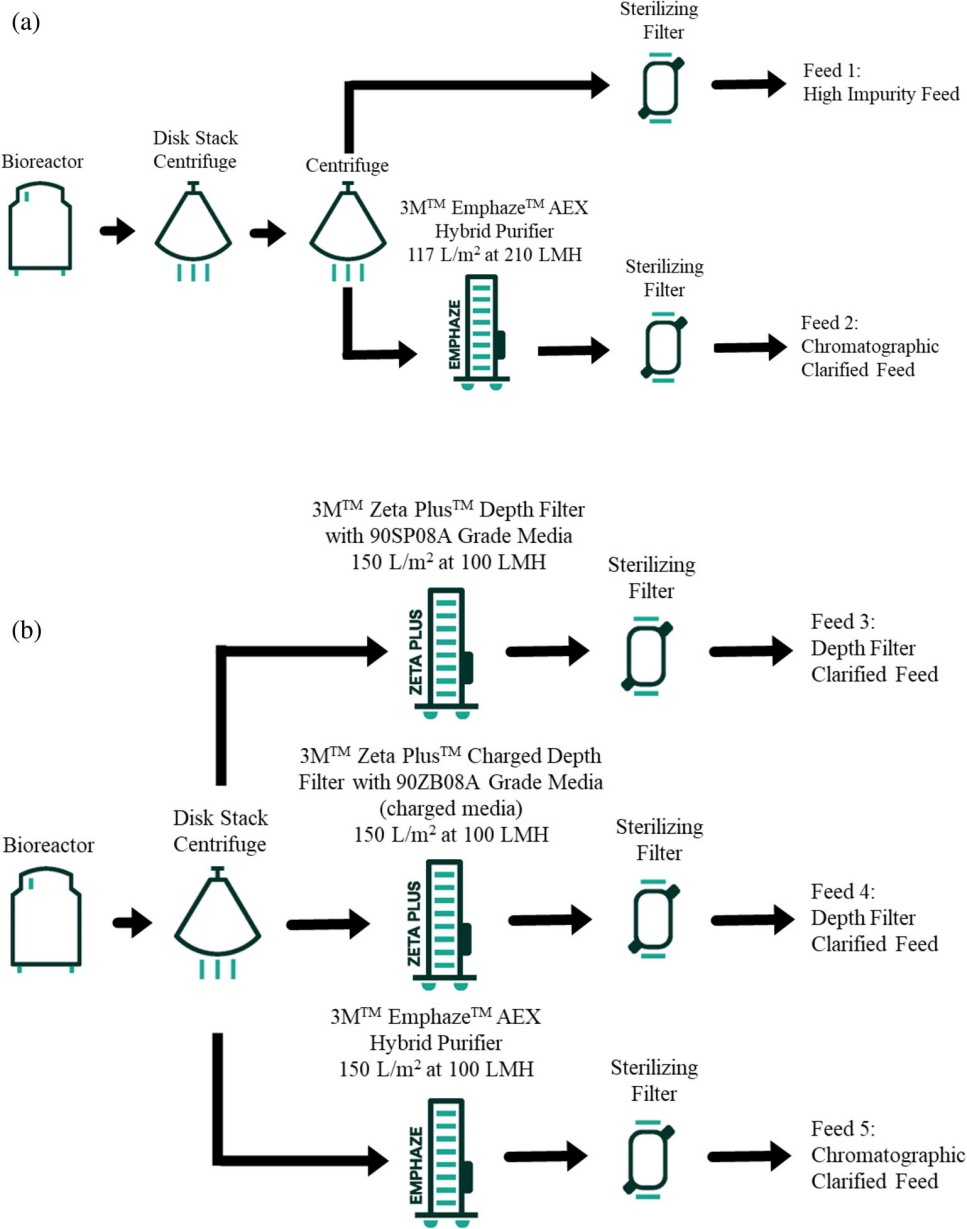
Clarification process for CHO cell culture 1 (a) and CHO cell culture 2 (b).

The two centrates were processed in different ways to generate a variety of feeds to cycle on the non‐resin Protein A columns. Figure 1 provides a summary of the clarification procedure for each culture. After thawing the centrate from cell culture 1 at 4°C, the fluid was centrifuged for 20 minutes at 17,000 rcf (Sorvall LYNX 6000 by Thermo Scientific) and sterile filtered, resulting in Feed 1. A portion of the sterile filtered fluid was processed using 3M™ Emphaze™ AEX Hybrid Purifier up to 117 L/m^2^ at 210 LMH and sterile filtered, resulting in Feed 2. The centrate generated from the frozen cell culture 2 was thawed at 4°C and processed by either 3M™ Zeta Plus™ Depth Filter with 90SP08A or 90ZB08A Grade Media or 3M™ Emphaze™ AEX hybrid purifier at 100 LMH up to a throughput of ~150 L/m^2^. After clarification, the fluids were sterile filtered (Feeds 3–5).

### 2.4 Protein A chromatography

The Protein A formats examined for rapid mAb purification were fiber‐based (Cytiva HiTrap Fibro® PrismA, 0.4 mL column volume) and membrane‐based (Gore® Protein Capture Device with Protein A, 1 mL column volume) scaffolds. The membrane‐based device has a reported dynamic binding capacity of 30 mg/mL of polyclonal IgG at 10% breakthrough with a residence time of 20 s. Similarly, the fiber‐based device has a reported dynamic binding capacity of 30 mg/mL of polyclonal IgG at 10% breakthrough but has a shorter residence time of 1.5–7.5 s. The cycling procedures for this study are reported in Tables 1 and 2 and contain a five‐step cycle for most of the Protein A chromatography cycling studies. In one example, a cleaning step with 0.5 M NaOH was performed. Mass loadings of the monoclonal antibody were 22.5–25 mg/mL and 25–28 mg/mL for the fiber‐based and membrane‐based devices, respectively, for Feeds 1–5. The entire elution volume was fractionated into tubes containing 200 μL neutralization buffer (2 M Tris base). A cleaning cycle was performed on several devices after the pressure limit or defined cycle number was reached (Table 2). Conditions were selected based on example procedures in the manufacturer's operating instructions manual associated with the products. Clarified solutions were sterile filtered before processing. Fiber and membrane Protein A devices were cycled up to 1.0 or 0.4 MPa, respectively, or until 200 cycles were reached.

**TABLE 1:** Process cycle for each format.

Step	Fiber (0.4 mL column volume or CV)	Membrane (1 mL column volume or CV)
Equilibration^a^	10 CV @ 16 mL/min	5 CV @ 3 mL/min
Load	2.5 mL @ 16 mL/min	7 mL @ 3 mL/min
Wash	20 CV @ 16 mL/min	6 CV @ 3 mL/min
Elution^b^	20 CV @ 16 mL/min	8 CV @ 3 mL/min
CIP^c^	20 CV @ 8 mL/min with 1 min hold	Did not use
Equilibration	30 CV @ 16 mL/min	8 CV @ 3 mL/min

^a^ Equilibration and Wash: 50 mM Tris–HCl, 150 mM NaCl, pH 7.5 ± 0.1.

^b^ Elution: 100 mM acetic acid, pH 3 ± 0.1.

^c^ CIP: 0.5 M sodium hydroxide.

**TABLE 2:** Cleaning cycle for each format.

Step	Fiber	Membrane
Equilibration^a^	10 CV @ 4 mL/min	4 CV @ 1 mL/min
50 mM NaOH	10 mL @ 4 mL/min	5 CV @ 1 mL/min
Equilibration	40 CV @ 4 mL/min	8 CV @ 1 mL/min
6 M guanidine HCl	10 mL @ 4 mL/min	5 CV @ 1 mL/min
Equilibration	30 CV @ 4 mL/min	12 CV @ 1 mL/min

^a^ Equilibration and Wash: 50 mM Tris–HCl, 150 mM NaCl, pH 7.5 ± 0.1.

### 2.5 Quantification Techniques

Protein quantification of mAb was performed according to prior procedures in Kohler et al. (2019). In summary, samples from cell culture and selected Protein A eluates were measured using a 0.1 mL (2.1 × 30 mm) POROS A 20 μm column on an Agilent HPLC system. A five‐point internal calibration standard in the sample matrix using a bind/elute buffer system was used to generate a standard curve. A standard curve was generated by using the area of the peaks, and sample areas were evaluated against the standard curve. HC‐DNA and HCPs in selected Protein A eluates and cell culture were analyzed using CHO‐specific qPCR and ELISA kits, respectively, according to manufacturer protocols (resDNASEQ™ Quantitative CHO DNA kit by Thermo Scientific and CHO HCP ELISA kit, 3rd generation, by Cygnus).

### 2.6 Turbidity analysis

Turbidity and acidified turbidity were measured using an ORION™ AQ4500 turbidity meter (Thermo Fisher Scientific) as described by prior work.^2^

## 3 RESULTS AND DISCUSSION

The objective of this study was to assess the impact of chromatographic clarification on convective Protein A cycling processes. 3M™ Emphaze™ AEX Hybrid Purifier technology during cell culture clarification generates filtrates with reduced levels of HC‐DNA and HCPs on Protein A resin columns, improving product purity and potentially extending the lifetime of the columns.^2^, ^7^, ^15^ This study further examines chromatographic clarification on emerging Protein A stationary phases by highlighting the impact on newer fiber‐ and membrane‐based Protein A scaffolds as examples. Clarified cell culture fluids (CCCF) produced for these cycling studies were characterized for turbidity, acidified turbidity, titer, HCP concentration, and HC‐DNA concentration and summarized in Table 3. Acidified turbidity, relating to the work of Koehler et al. (2019), is a technique used to indicate HC‐DNA breakthrough post‐clarification by precipitation of chromatin‐DNA complexes. A higher acidified turbidity compared to the native turbidity is indicative of HC‐DNA breakthrough. Clarified feeds contained HCP and HC‐DNA concentrations from 0.17 to 0.62 mg/mL and <0.06 to 31,696 ng/mL, respectively. 3M™ Emphaze™ AEX Hybrid Purifier processed feed showed significantly lower HCP and HC‐DNA concentrations while maintaining greater than 95% product recovery under the conditions tested.

**TABLE 3:** CCCF feed for Protein A chromatography cycling studies.

Cell culture	Feed no.	Process	Turbidity (NTU)	Acidified turbidity (NTU)	mAb titer (mg/mL)	HCP (ng/mL)	HC‐DNA (ng/mL)
1	Feed 1	Centrate (control)	35.1	1,180	3.55	627,510	31,696
	Feed 2	3M™ Emphaze™ AEX Hybrid Clarifier	6.3	5.0	3.61	442,925	<0.06 (LOQ)
2		Centrate (control)		543	4.07	325,714	36,443
	Feed 3	3M™ Zeta Plus™ Depth Filter with 90SP08A Grade Media	14.5	269	4.01	309,388	18,176
	Feed 4	3M™ Zeta Plus™ Depth Filter with 90ZB08A Grade Media	8.8	103	4.02	312,050	10,141
	Feed 5	3M™ Emphaze™ AEX Hybrid Clarifier	4.6	3.6	4.02	169,894	<0.06 (LOQ)

*Note*: All processed fluids sterile filtered with 0.2 μm membrane.

### 3.1 Lifetime analysis with challenging feed

**FIGURE 2:**
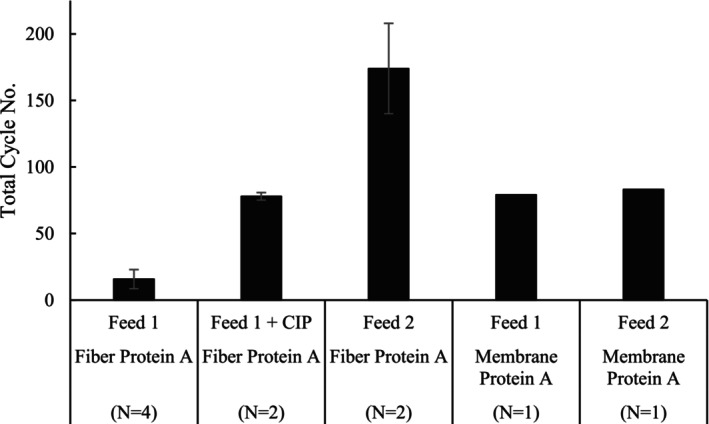
Total number of cycles run until reaching the pressure cutoff (1 and 0.4 MPa for fiber Protein A and membrane Protein A devices, respectively).

Fiber or membrane‐based Protein A devices were cycled up to three different ways with either Feed 1 or Feed 2 derived from cell culture 1 up to 200 cycles or until a defined pressure limit for each device was reached: (1) Feed 1 cycled with no CIP (clean‐in‐place) step, (2) Feed 1 cycled with a CIP step included (only fiber‐based Protein A), and (3) Feed 2, chromatographically clarified, feed with no CIP step. Figure 2 summarizes the number of cycles run before a set pressure limit of each rapid purification Protein A device was reached. This challenging scenario pushes the boundaries of use for these devices as CIP with sodium hydroxide (NaOH) use is a standard practice in biopharmaceutical manufacturing for cleaning and sanitizing Protein A affinity resin and rapid formats and is typically used during every cycling step.

Results demonstrated that feed clarified using functional AEX fiber approach prior to cycling had a significant impact on increasing the lifetime of the fiber‐based Protein A device. Fiber‐based Protein A was able to be cycled 150–198 times with a chromatographically clarified feed (Feed 2) compared to 7–23 times with a centrate‐only feed (Feed 1). Additionally, an increase in fiber‐based Protein A cycling lifetime was observed using AEX fiber clarified feed (Feed 2) compared to cycling Feed 1 containing a 0.5 M NaOH cleaning step after elution for each cycle run. Removal of process impurities, such as nucleic acids and proteins, before cycling on these rapid mAb purification fiber‐based devices suggested chromatographic clarification had a more pronounced effect by reducing the pressure buildup on the scaffold, leading to an increase in lifetime than incorporating a NaOH CIP step during every cycle. A combination of cleaning procedures, including a regeneration or cleaning step, with chromatographic clarification methods may extend the lifetime further but requires additional time to the purification process and Cost of Goods to accomplish.

3M™ Emphaze™ AEX Hybrid Purifier was shown to significantly reduce HC‐DNA and chromatin‐related impurities comprising a particle size of ~0.1 μm.^2^ However, the pore size of this fiber‐based Protein A product reported a measured pore size diameter of 0.25 ± 0.03 μm.^16^ One could argue the pore sizes are large enough to pass through the HC‐DNA and associated complexes, but Protein A has been shown to be capable of indirectly binding chromatin through histones and interfering with ligand access for antibody binding.^17^ One study indicated endonuclease‐treated CCCF processed by depth filtration reduced the rate of pressure increase inside a fiber‐based Protein A device by two‐fold compared to CCCF without endonuclease treatment, suggesting HC‐DNA contributes to fouling of the fiber‐based Protein A media, but endonuclease treatment did not eliminate it completely.^6^ As this study does not intend to identify the root cause of fouling, additional studies should be considered to elucidate the mechanism.

**FIGURE 3:**
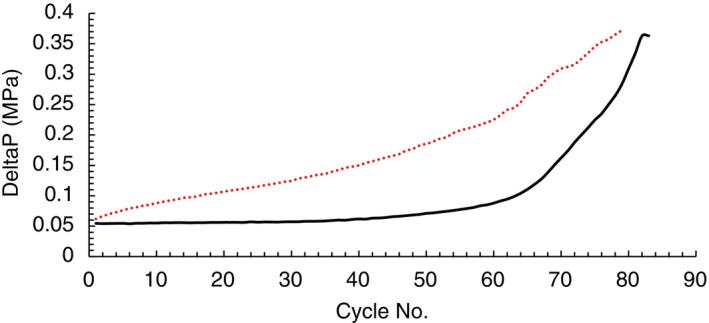
Maximum delta pressure during the elution phase of each cycle purifying mAb from Feed 1 (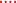) or Feed 2 (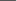) using membrane‐based Protein A.

As for the membrane‐based devices tested, there was only a marginal increase, if any, in lifetime observed with the chromatographically processed feed cycled. Since a similar number of cycles were observed, an experiment including a CIP step with NaOH was not incorporated. Additional studies may be useful to elucidate the effect of implementing a sodium hydroxide treatment in combination with chromatographic clarification on lifetime and impurity removal. Figure 3 shows a steady pressure increase when processing Feed 1 (high impurity feed) over the course of cycling use, but Feed 2 (feed treated using 3M™ Emphaze AEX Hybrid Purifier‐treated feed) when cycled exhibited a low pressure to about 60 cycles before pressure elevated quickly over the next 23 cycles.

The mechanism of fouling appears to differ from this membrane compared to the previously described fiber‐based Protein A chromatography examined in this study. Even though significant levels of HC‐DNA were removed by functional AEX fiber during clarification, host cell protein concentration in Feed 2 was reduced by 29.4% but still contained >120,000 ppm HCP. Pathak and Rathore^18^ concluded, in their research, fouling of Protein A resin through build‐up of proteins inside the resin as intra‐particle porosity was reduced in resin cycled 100 times with cell culture feed.^18^ The membrane‐based Protein A device tested here is reported to be comprised of porous silica bead, containing a pore size of ~100 nm in diameter, embedded in a ePTFE matrix and supporting data suggests Protein A ligands are immobilized in pores of the silica beads.^16^, ^19^ One study demonstrated the dynamic binding capacity of the composite membrane tested here was dependent on flow rate suggesting a diffusion‐related transport like resin but contains flow distribution and permeability properties allowing for flow‐independent elution volumes.^16^ One possible explanation for fouling could be the association of lipophilic proteins to mAb after mAb binding to the Protein A ligand induced conformational changes where a low pH elution desorbed these complexes which were caught within the resin pores.^20^ Other studies demonstrated the presence of proteins, including IgG and associated fragments, when extracting proteinaceous species from resin using SDS.^21^, ^22^ Another possible factor contributing to the fouling could be the PTFE portion of membrane composite. There is a general understanding that hydrophobic surfaces, such as PTFE, tend to absorb proteins more strongly than hydrophilic surfaces.^23^, ^24^ The influence of HCP on fouling of this membrane may be only one part of a more complex aging mechanism encompassing product‐ and process‐related impurities, possibly similar to resin given the porous particles associated to this membrane.^16^, ^17^, ^25^, ^26^

**FIGURE 4:**
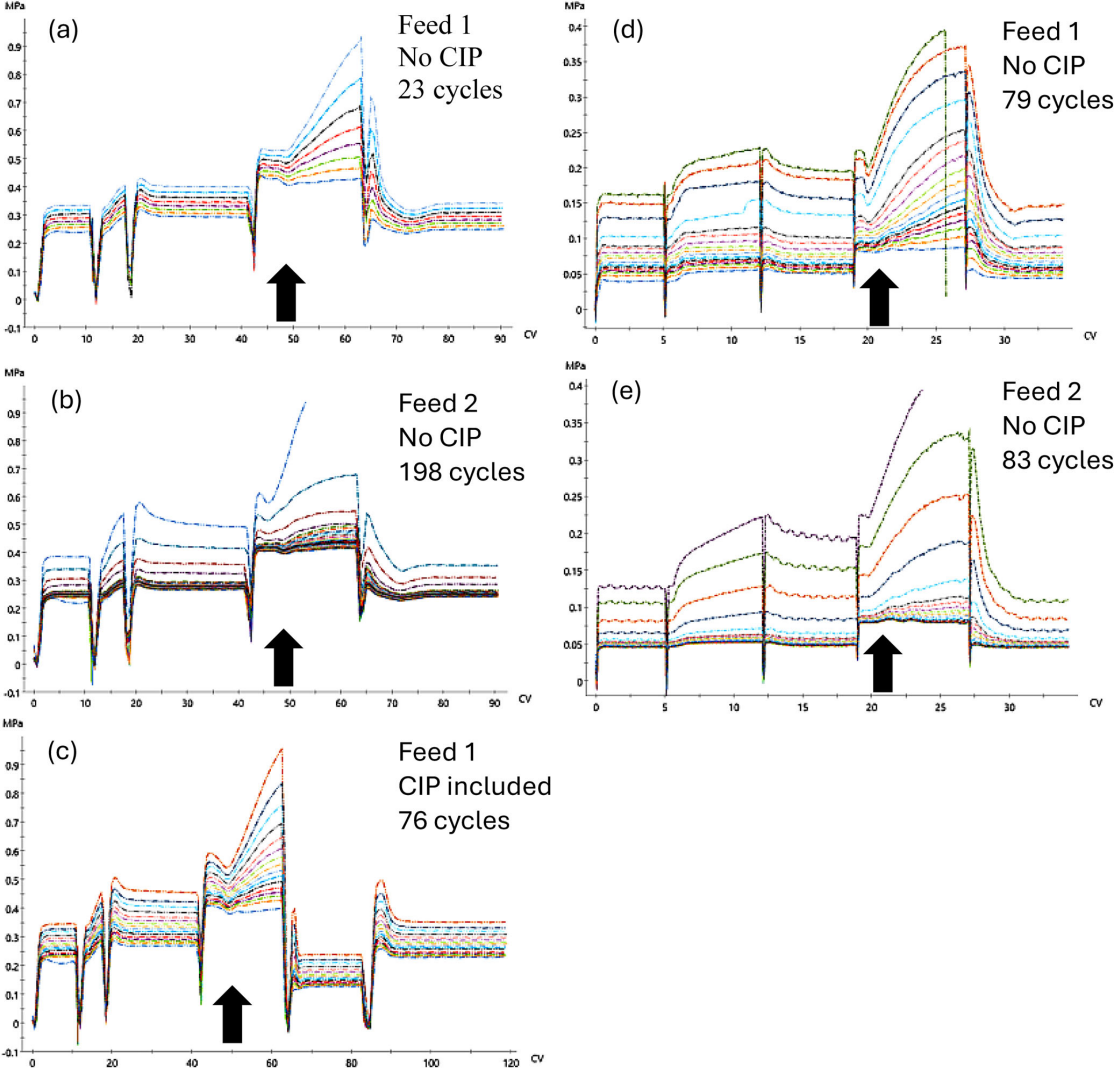
Example chromatograms from Table 4 showing precolumn pressure curves of selected cycles from fiber‐based (a–c) and membrane‐based (d, e) Protein A devices over the course of cycling. Fiber‐based Protein A cycled 23 times with Feed 1 (a), fiber‐based Protein A cycled 198 times with Feed 2 (b), fiber‐based Protein A cycled 76 times with Feed 1 including a NaOH CIP step (c), membrane‐based Protein A cycled 79 times with Feed 1 (d), and membrane‐based Protein A cycled 83 times with Feed 2 (e). Arrows indicate elution step. Y‐axis is the precolumn pressure in megapascal (MPa) and X‐axis is the column volume (CV) of the process run.

The major purpose of having an extended low pH elution step was to assess maximum product and impurity recovery. During the extended low pH elution, we observed the highest pressure increase for both the fiber and membrane Protein A media during this step over the course of the cycling study. Figure 4 shows pressure curves from the elution phase during selected cycle steps. Upon neutralization in the equilibration buffer, the pressure decreased to levels close to the initial cycle pressure. Pressure continues to build in each cycle after applying more cell culture fluid followed by low pH elution on the Protein A stationary phase. Transient pressure increase during the low pH elution of mAbs has been reported using Protein A resins, and one study suggested liquid–liquid phase separation produced turbidity contributing to the pressure increase.^27^ However, we observed from the stationary phases tested in this study that the pressure increase does not appear transient but reversible upon equilibration with 50 mM Tris–HCl, 150 mM NaCl, pH 7.5 buffer. This may indicate continued build‐up on the surface or in the pores of the affinity capture functionalized media layers by cell culture media components, products, or impurities over the course of cycling, which are impacted by acid‐induced changes leading to increased pressure not limited to the type of stationary phase.^17^, ^18^, ^28^, ^29^, ^30^

**FIGURE 5:**
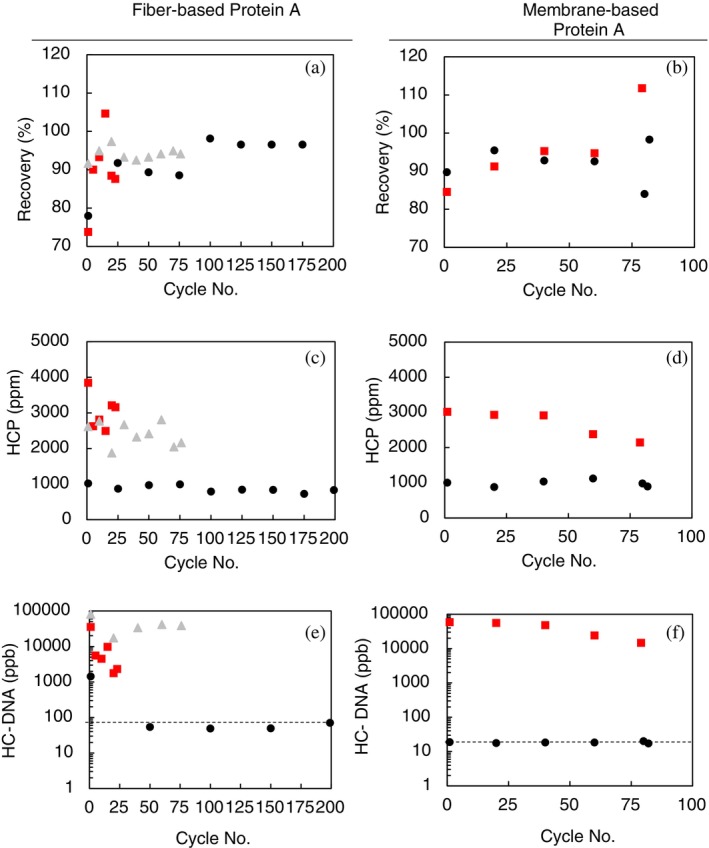
Process metrics for fiber‐based Protein A (a, c, e) and membrane‐based Protein A (b, d, f) devices cycled with Feed 1 (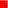), Feed 1 with CIP step (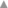), and Feed 2 (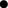). Product recovery (a, b), host cell protein concentration (c, d), and host cell DNA concentration (e, f) were measured from selected eluates. Limit of quantification for DNA indicated by dashed black line.

Process metrics were analyzed as part of this study beyond the impact of lifetime based on pressure and to identify differences in performance based on feed quality. As shown in Figure 5, Protein A eluates were examined for monoclonal antibody product recovery, host cell protein concentration, and HC‐DNA concentration. In general, early cycle product recovery was below 90% and stabilized shortly after the first few runs. However, there appeared to be some variation in product recovery toward the later cycle runs, where pressure was right near the pressure limit of the device. An exception for this was the fiber‐based device with cycling untreated feed with a CIP step in each cycle that showed consistent product recovery (>90%) across the time it was used. Since product recovery was near 90% and did not appear to reduce significantly in the later cycle runs as the pressure increased, modification of processing techniques could play a role in increasing lifetime use of these devices. Decreasing the flow rate may help avoid over‐pressurizing as the devices approach their respective pressure limits and potentially increase the number of capture cycles. One drawback could be a loss in productivity during the last stage cycles as decreasing the flow rates would increase processing times to maintain an operational pressure.

HCP impurity levels were consistent in Protein A eluates from devices cycling the chromatographically clarified feed. Normalized HCP concentration (ppm; ng HCP per mg mAb) was ~1,000 ppm in eluates from devices cycled with AEX fiber processed feeds, but devices cycled with feeds clarified with membrane filtration (with and without CIP) varied between 1,900 and 3,900 ppm for both Gore Protein Capture and Fibro PrismA devices.

In general, HC‐DNA levels in Protein A eluates for devices cycled with 3M™ Emphaze™ Hybrid Purifier clarified feed were consistently below the limit of quantification. However, the first cycle for Fibro PrismA did have a concentration of HC‐DNA of 1,400 ppb (ppb: ng DNA per mg mAb) but stabilized in later cycles. Eluates from devices cycled with Feed 1 (with and without CIP) varied with HC‐DNA concentration across the cycles, ranging from ~15,000–60,000 ppb with membrane‐based Protein A elutes and 1,800–80,000 ppb with fiber‐based Protein A eluates.

**FIGURE 6:**
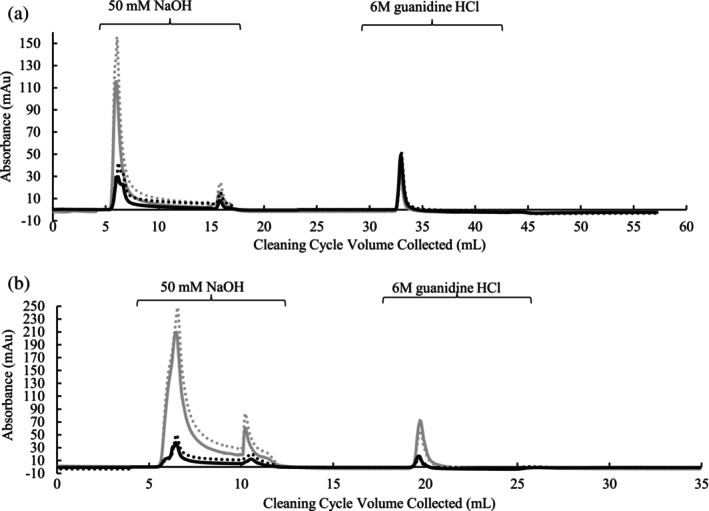
Overlays of UV absorbance spectra at 260 nm (dots) and 280 nm (solid lines) of fiber‐based and membrane‐based Protein A devices cleaned with 50 mM NaOH and 6 M guanidine HCl. (a) Fiber‐based devices from Lot 1 cycled 7 times with Feed 1 (gray) or 150 times with Feed 2 (black). (b) Membrane‐based devices cycled 79 times with Feed 1 (gray) or 83 times with Feed 2 (black).

A cleaning protocol was run after the device pressure limit was reached to clean and strip off impurities bound to Protein A scaffol. UV absorbance spectra (Figure 6) show larger peak areas from devices cycled with Feed 1 (high impurity feed) times compared to the devices cycled with Feed 2 (AEX fiber processed feed). This demonstrates AEX chromatographically clarified processed feed resulting in lower column‐bound impurities eluted from both fiber‐based and membrane‐based Protein A stationary phases during the cycle. Since lower concentrations of impurities were eluted from this media, this may provide an opportunity to reduce concentrations of NaOH regeneration or potentially eliminate other cleaning steps resulting in reduced processing times.

### 3.2 The effect of clarification on cycling performance

For this portion of the study, several different clarification approaches were chosen to understand the impact on these protein‐A technologies, as described in Figure 1. A lower charged depth filter technology (3M™ ZetaPlus™ 90SP08A Depth Filter) was compared to a similarly sized pore fine grade depth filter with a higher charge (3M™ ZetaPlus™ 90ZB08A Depth Filter). In addition, the chromatographic clarification approach was also evaluated using the 3M™ Emphaze™ AEX Hybrid Purifier. Fiber or membrane Protein A devices were cycled with either a depth filter or functional fiber clarified feed up to 200 cycles. The pressure was monitored over each cycle, and eluates were examined for impurity level and product recovery.

**FIGURE 7:**
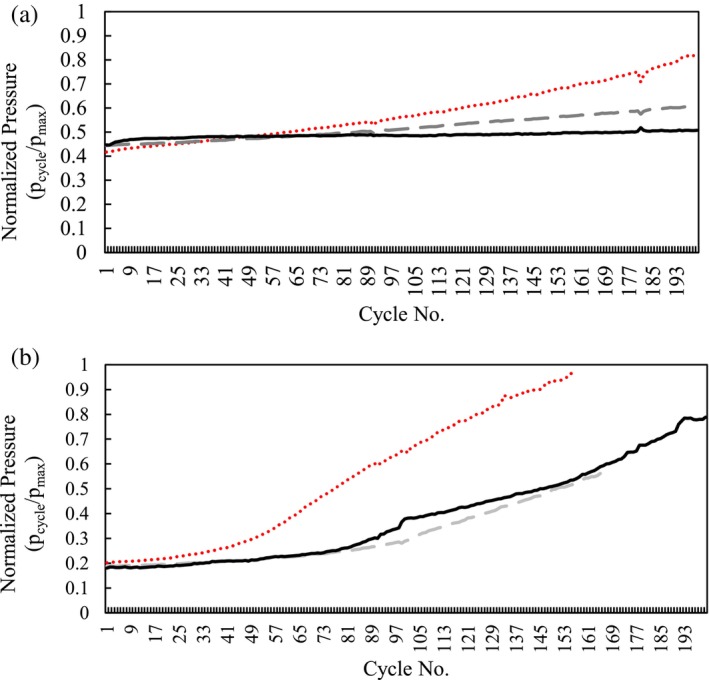
Normalized precolumn pressure during the elution phase of purifying mAb by cycling (a) fiber or (b) membrane‐based Protein A device with Feed 3 (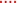), Feed 4 (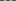), and Feed 5 (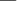).

Devices containing fiber‐based Protein A were able to process up to 200 cycles, the maximum cycles tested. As presented in Figure 7a, an increase in pressure indicated device fouling and appears to be correlated with the turbidity and acidified turbidity measurements (Table 4). Acidification of clarified solutions has been previously shown to be correlated with the precipitation of chromatin and HC‐DNA impurities.^2^ This could be one explanation why we see higher‐pressure increases were noticed during the low pH elution phase, as previously mentioned. Higher concentrations of HC‐DNA in Feeds 3 and 4 from depth filtration may have led to more nonspecific binding to the stationary phases and potentially precipitated during low pH elution. Thus, clarified feeds with higher acidified turbidity measurements may lead to faster fouling for these fiber‐based Protein A devices.

**TABLE 4:** Comparison of pressure change (Cycle 1 vs. Cycle 200) and acidified feed turbidity for Cytiva Fibro™ PrismA.

Clarification train	Feed	Turbidity (NTU)	Acidified turbidity (NTU)	Increase in precolumn pressure over 200 runs (%)
3M™ Zeta Plus™ Depth Filter with 90SP08A Grade Media	3	14.5	269	96.4
3M™ Zeta Plus™ Depth Filter with 90ZB08A Grade Media	4	8.8	103	36.9
3M™ Emphaze™ AEX Hybrid Purifier	5	4.6	3.6	13.5

For the membrane‐based Protein A tested, only 156 cycles were reached for the device cycled with lower charge 3M™ Zeta Plus™ 90SP08A Grade Filter Media clarified feed before the pressure limit was reached, while 200 cycles were reached with 3M™ Emphaze™ AEX Hybrid Purifier clarified feed. The device purifying mAb from the higher charge 3M™ Zeta Plus™ 90ZB08A Grade Filter Media (charged media) clarified feed stopped at 165 cycles due to insufficient volumes needed to complete 200 cycles. However, Figure 7b indicates a similar pressure increase with devices processing fluids clarified by 3M™ Emphaze™ AEX hybrid purifier and 3M™ Zeta Plus™ Depth Filter with higher charge.

**FIGURE 8:**
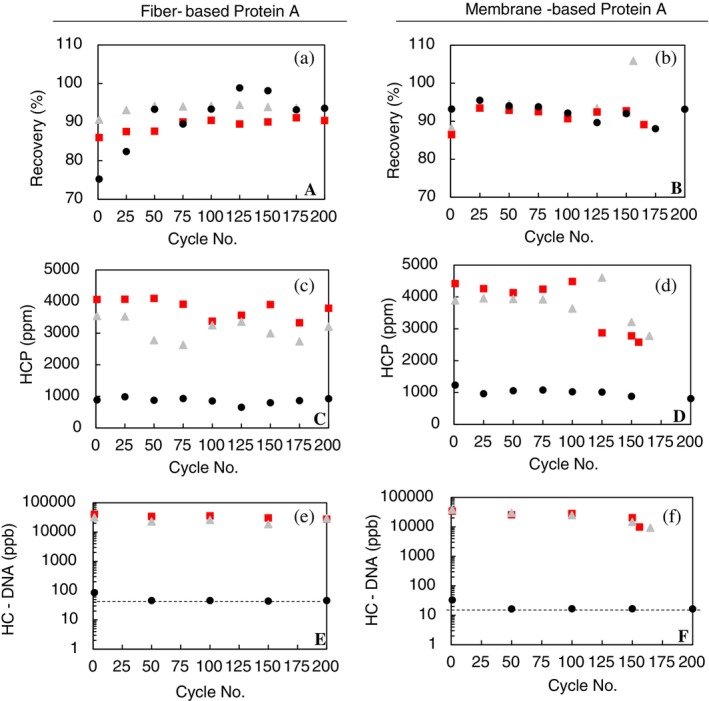
Process metrics for fiber‐based Protein A (a, c, e) and membrane‐based Protein A (b, d, f) devices cycled with Feed 3 (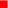), Feed 4 (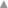), and Feed 5 (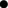). Product recovery (a, b), host cell protein concentration (c, d), and host cell DNA concentration (e, f) were measured from selected eluates. Limit of quantification for DNA indicated by dashed black line.

Protein A eluates were analyzed for mAb product recovery, HCP concentration, and HC‐DNA concentration (Figure 8). In general, early cycle product recovery was below 90% and stabilized above 90% in later runs.

HCP impurity levels were consistent in Protein A eluates from devices cycling chromatographically clarified feed. Normalized HCP concentration was approximately 1000 ppm in eluates from devices cycled with AEX fiber clarified feeds, but devices cycled with low charge and high charge depth filters were 2600 and 4600 ppm for both membrane and fiber Protein A devices, respectively. Interestingly, the HCP concentration in the eluates from membrane Protein A was relatively consistent up to about 100 cycles before the concentrations started to be reduced in the eluates. This led to cycle‐to‐cycle variation across the lifetime, whereas eluates of devices cycled with the AEX clarified feed demonstrated lower and consistent HCP concentrations. A similar trend was shown previously with cell culture 1.

In general, HC‐DNA levels in Protein A eluates for devices cycled with AEX chromatographic clarified feed were consistently below the limit of quantification. However, the first cycle for fiber and membrane‐based Protein A did have a concentration of HC‐DNA of 85 and 33 ppb, respectively, but stabilized in later cycles. Eluates from devices cycled with the low charge and high charged depth filter processed feeds varied with HC‐DNA concentration across the cycles, ranging from ~9,000 to 38,000 ppb with membrane Protein A processed eluates and 18,000 to 40,000 ppb with fiber Protein A processed eluates.

## 4 CONCLUSIONS

Chromatographic clarification using AEX fiber chromatography combined with rapid cycling of non‐resin stationary phases of Protein A chromatography devices demonstrated consistent and low HCP and HC‐DNA concentrations in eluates over the use of these devices, leading to increased product purity. In this study, Protein A eluate HCP levels were consistent around 1,000 ppm, while HC‐DNA concentrations were routinely below levels of quantification in most, if not all, cycle eluates. This study demonstrates reduced batch‐to‐batch variability, suggesting more consistent impurity profiles regardless of the Protein A stationary phase being used.

AEX chromatographic clarifiers may increase the lifetime of fiber or membrane‐based Protein A chromatography formats through the removal of impurities. Fiber‐based Protein A was cycled at least 8X more with chromatographically clarified feed (Feed 2) compared to a centrate‐only feed (Feed 1). Additionally, we observed a greater than 2X increase in fiber‐based Protein A cycling lifetime using AEX chromatographic clarified feed (Feed 2) compared to Feed 1 cycling containing a 0.5 M NaOH cleaning step after elution for each cycle run.

The operational lifespan of Protein A chromatography devices can be influenced by several factors, including pressure limitations and yield loss. Ligand degradation, which may occur through mechanical or chemical stresses, can diminish the binding capacity of Protein A stationary phases, leading to a product yield loss. Product yield remained relatively consistent in this study, and no significant drop in binding capacity was observed. This result suggests that ligand degradation was not the primary mechanism limiting device lifetime. We hypothesize that fouling played a significant role in limiting the lifespan of the devices tested in this study. The devices may have fouled as an accumulation of impurities blocked the pores in the matrices, leading to increased pressure within the devices and eventually pushing them past the recommended operating pressure.

This study demonstrated the importance of chromatographic clarification in maximizing utilization of the new generation of rapid cycling Protein A capture chromatography technologies. The removal of host cell‐related impurities and particulates before processing and cycling rapid Protein A formats improved product purity post Protein A and increased, in some cases significantly, the lifetime of these capture chromatography devices.

## AUTHOR CONTRIBUTIONS

**Andrew Vail:** Conceptualization; data curation; formal analysis; funding acquisition; investigation; methodology; writing – original draft. **David Chau:** Conceptualization, methodology, writing – review and editing. **Jennifer Heitkamp:** Conceptualization, methodology, formal analysis, writing – review and editing. **Alexei Voloshin:** Conceptualization; methodology; supervision; writing – review and editing.

## CONFLICT OF INTEREST STATEMENT

All authors were employees of Solventum (formerly 3M Health Care), the corporation that developed and produced 3M™ Emphaze™ AEX Hybrid Purifier and 3M™ Zeta Plus™ depth filters, at the time the work was performed.

## Data Availability

The data that support the findings of this study are available from the corresponding author upon reasonable request.
